# Clinical and histologic features of botryoid odontogenic cyst: a case report

**DOI:** 10.1186/1752-1947-4-260

**Published:** 2010-08-10

**Authors:** Vitor H Farina, Adriana AH Brandão, Janete D Almeida, Luiz AG Cabral

**Affiliations:** 1 Department of Biosciences and Oral Diagnosis, São José dos Campos Dental School, São Paulo State University-UNESP, São José dos Campos, São Paulo, Brazil

## Abstract

**Introduction:**

The lateral periodontal cyst, as the name implies, occurs on a lateral periodontal location and is of developmental origin, arising from cystic degeneration of clear cells of the dental lamina. A botryoid odontogenic cyst is considered to be a rare multilocular variant of a lateral periodontal cyst.

**Case presentation:**

We report the clinical and histopathologic features of a rare case of botryoid odontogenic cyst found in an edentulous area corresponding to the right lower canine of a 64-year-old African-American woman. A multilocular radiolucency was observed, and surgical removal of the lesion revealed a nodule of rubber-like consistency measuring about 1.5 cm in diameter. Cross-sectioning of the nodule showed that it consisted of various cystic compartments. Histologically, various voluminous periodic acid-Schiff-negative clear cells randomly distributed throughout the cystic epithelium were observed, as well as cell layers showing thickenings generally formed by oval, sometimes entangled plaques. The capsule consisted of fibrous connective tissue and showed rare and discrete foci of a perivascular mononuclear inflammatory infiltrate and reactive bone-tissue fragments. The final diagnosis was botryoid odontogenic cyst.

**Conclusion:**

We provide data that allow the reader to establish the differences between botryoid odontogenic cyst, glandular odontogenic cyst, and lateral periodontal cyst, helping with the differential diagnosis. The reader will have the opportunity to review botryoid odontogenic cyst clinical and histopathologic features, including treatment.

## Introduction

Botryoid odontogenic cyst (BOC) was originally described in 1973 by Weathers and Waldron [1] as an intraosseous lesion characterized by a macroscopic and microscopic multilocular growth pattern, resembling a bunch of grapes (from the Greek word *botrios*). BOC is considered to be a variant of a lateral periodontal cyst [2]. Greer and Johnson [3], reviewing 10 cases of BOC, observed that nine of the lesions were located in the mandible, mainly the anterior region, and one in the maxilla, also in the anterior region. Radiologically, eight cases were characterized by unilocular radiolucencies, and two, by multilocular radiolucencies. The mean age of the patients was 46 years. Three cases were recurrences, one occurring eight years, and two, 10 years after treatment. Histologically, non-keratinized squamous epithelium consisting of a few cell layers was observed, which showed plaque-like thickening in seven cases. Zones of clear cells scattered throughout the epithelium and containing periodic acid-Schiff (PAS)-positive material were observed in all 10 cases. In four cases, a discretely hyalinized zone underlying the basal epithelial layer was noted. All 10 lesions were characterized by the presence of multiple cystic compartments.

In a study evaluating 66 cases of BOC, Ramer and Valuri [4] found a slight predominance in women (53%) over men (47%), with 70% of the cases occurring in white individuals and 30%, in black individuals. Heikinheimo *et al. *[5] reported a case of BOC with multiple recurrences that had occurred over a period of nine years, which led the authors to propose more-radical surgical intervention in cases of BOC. Manor *et al. *[6] stated that BOC frequently shows a lobulated radiographic pattern similar to that of glandular odontogenic cysts and, therefore, the latter should be included in the differential diagnosis. However, these cysts are characterized by the presence of salivary gland-like structures in the lining epithelium, an uncommon histologic finding in BOC [7].

## Case presentation

A 64-year-old African-American woman presented at our outpatient clinic with a two-year history of asymptomatic enlargement in the right anterior region of the edentulous mandible.

**Figure 1:**
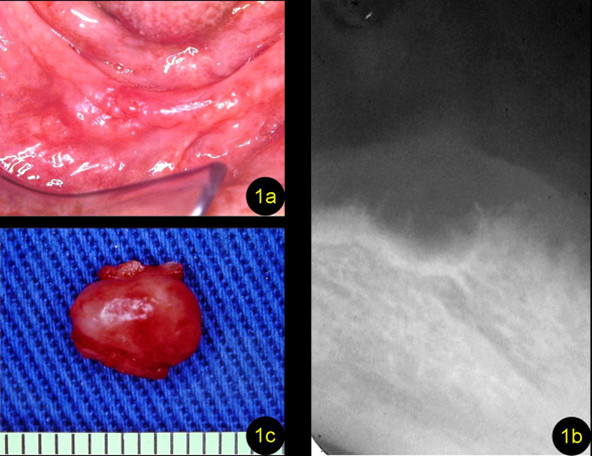
**Details of the asymptomatic enlargement**. **(a) **Expansion located in the region corresponding to the right lower canine lined with intact mucosa and presenting a multi-lobulated surface. **(b) **Occlusal radiograph showing a well-delimited multilocular radiolucent lesion. **(c) **Nodule measuring about 1.5 cm in diameter; it was removed surgically.

Clinical examination revealed an expansion, measuring approximately 2 cm in the major diameter and located in the region corresponding to the right lower canine, which was of firm consistency on palpation, lined with intact mucosa, and presented a multi-lobulated surface (Figure 1a). Occlusal radiography showed a well-delimited multilocular radiolucency in the region of expansion (Figure 1b). On the basis of these data, the differential diagnosis of a cyst of odontogenic origin was established.

**Figure 2:**
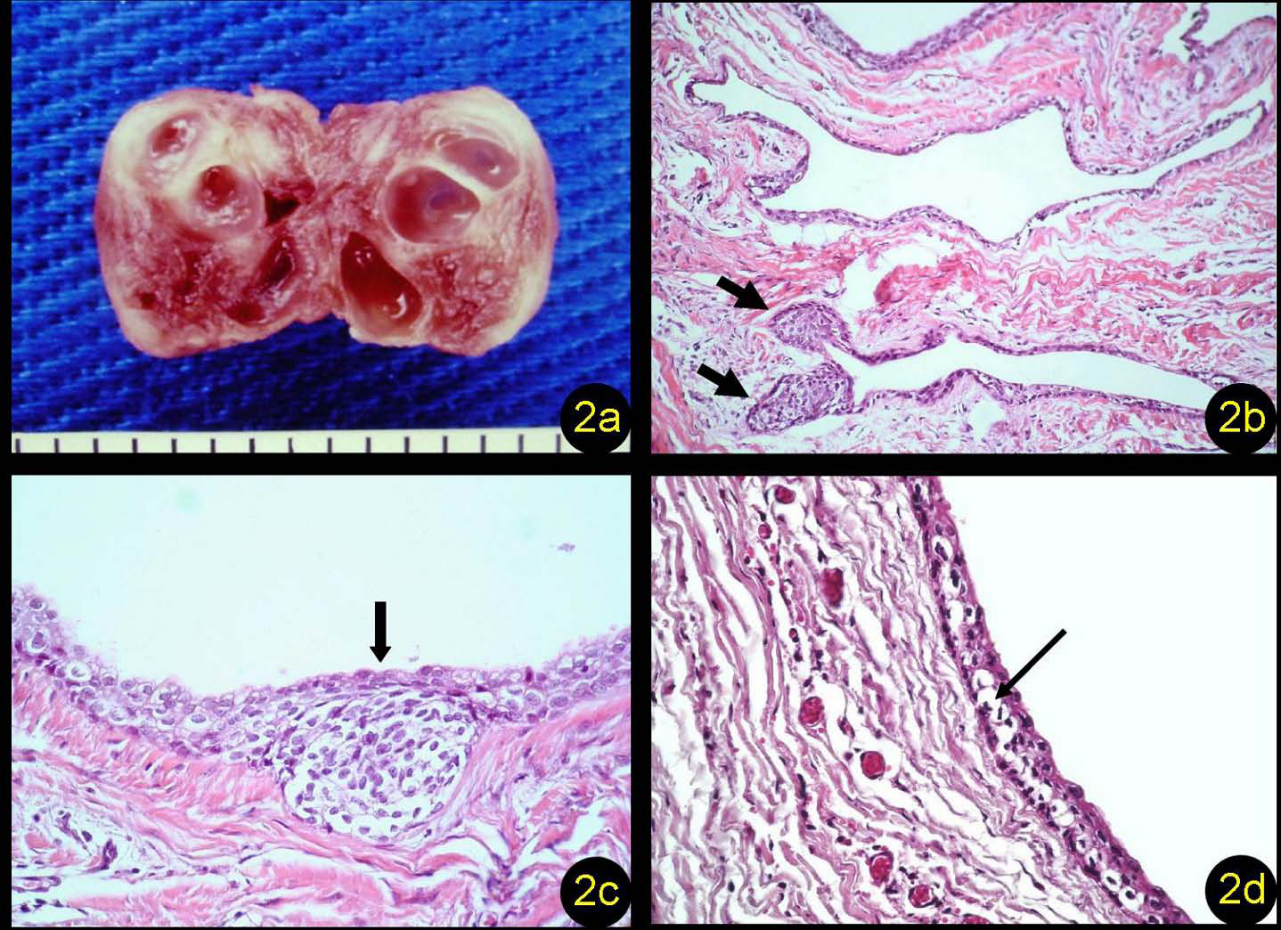
**Studies of the cyst**. **(a) **Cross-sectioned nodule showing various cystic compartments. **(b) **Multiple cystic cavities lined with thin pavement epithelium with thickened areas (arrows) (H&E, 200×). **(c) **Plaque-like focal thickening in the epithelial lining of the cyst (arrow) (H&E, 400×). **(d) **Cystic cavity showing PAS-negative voluminous clear cells in the lining epithelium (arrow) (PAS, 400×).

Surgical removal of the lesion, which was easily separated from the surrounding bone, revealed a nodule of rubber-like consistency measuring about 1.5 cm in diameter (Figure 1c). Cross-sectioning of the nodule showed that it consisted of various cystic compartments (Figure 2a). The surgical specimen was sent for histopathologic analysis, which showed the presence of multiple cystic cavities of variable size and shape and thin walls lined with non-keratinized stratified pavement epithelium of variable thickness; the epithelium either consisted of a few cell layers or showed thickenings generally formed by oval, sometimes entangled, plaques (Figure 2b and 2c). In addition, various voluminous PAS-negative clear cells randomly distributed throughout the cystic epithelium were observed (Figure 2d). The capsule consisted of fibrous connective tissue and rare and discrete foci of a perivascular mononuclear inflammatory infiltrate and reactive bone-tissue fragments. The final diagnosis was BOC.

## Discussion

The case of BOC reported in the present study was characterized clinically by an asymptomatic nodule located in the right anterior region of the mandible, and radiographically, by a multilocular radiolucent lesion, reflecting the macroscopic aspect of the surgical specimen, a phenomenon not always observed in BOC because this type of cyst tends to present a unilocular radiographic aspect [4]. Conversely, the multilocular radiographic aspect is very frequent in cases of glandular odontogenic cysts [6]; however, these cysts show glandular ductlike structures in the lining epithelium, not observed in this case.

In the present case, the microscopic observation of multiple cystic cavities lined with non-keratinized stratified pavement epithelium consisting of a few cell layers with focal entangled thickenings, the presence of voluminous clear cells in the epithelium, and a thin connective tissue capsule with a few inflammatory cells, combined with the clinical and radiographic findings, defined the diagnosis of BOC, as also reported by Falcone *et al. *[8]. The fact that the clear cells present in the epithelium were not stained with PAS indicates that they did not contain glycogen or is just an example of the vagaries of histochemical staining procedures. Negative findings may well be due to tissue handling or other technical details. This finding is in contrast to the results of Greer and Johnson [3] and Gurol *et al. *[9], who observed staining of these cells with PAS. Greer and Johnson [3] reported a similarity between these clear cells rich in glycogen, frequently observed in the epithelium of BOC, and dental lamina cells, suggesting that the dental lamina is one of the possible origins of BOC. However, these cells do not seem to exert any influence on the biologic behavior of the cyst. The diagnosis of BOC should not be discarded in cases of negative PAS staining when all other histologic features are present.

With respect to the similarity between BOC and lateral periodontal cysts, Machado de Souza *et al. *[10] stated that BOC are distinguished from the latter by their larger size, which, associated with the multilocular characteristic of BOC, increases the possibility of recurrence because complete surgical removal becomes more difficult.

Üçok *et al. *[2] reported that the risk of recurrence of BOC is similar to that of keratocystic odontogenic tumors, but that the former shows less aggressive behavior. According to Ramer and Valuri [4], BOC is not an aggressive lesion, and recurrences are the result of conservative surgical treatment (enucleation). The authors observed that 10 of 13 recurrences studied presented a multilocular radiographic aspect and suggested more-frequent postoperative follow-up visits in these cases.

The case reported here has been followed up for eight years. So far, no clinical or radiographic evidence characterizes recurrence of the lesion, indicating that the lesion was removed *in toto*. However, follow-up for a longer period is necessary to ensure the success of surgical treatment in this case of BOC.

## Conclusion

Recurrences of multilocular radiolucent lesions of BOC are common. A non-conservative surgical removal is the only effective treatment for this kind of lesion.

## Competing interests

The authors declare that they have no competing interests.

## Authors' contributions

JDA and LAGC analyzed and interpreted the patient data and performed the surgical procedures. AAHB performed the histologic examination and photographs. VHF was a major contributor in writing the manuscript. All authors read and approved the final manuscript.

## Consent

Written informed consent was obtained from the patient for publication of this case report and accompanying images. A copy of the written consent is available for review by the Editor-in-Chief of this journal.
